# Biomimetic ECM collagen hydrogel induces chondrogenic differentiation of BMSCs by activating autophagy through POU5F1-mediated AMPK/mTOR signaling pathway

**DOI:** 10.7150/thno.126710

**Published:** 2026-03-30

**Authors:** Xiao Ru, Zhongwen Yu, Jun Li, Fuben Xu, Guojie Xu, Junqi Xie, Simeng Yu, Pan Hu, YuanYuan Liu, Li Zheng, Jinmin Zhao, Zhenhui Lu

**Affiliations:** 1 Guangxi Engineering Center in Biomedical Materials for Tissue and Organ Regeneration, Collaborative Innovation Centre of Regenerative Medicine and Medical BioResource Development and Application, Guangxi Key Laboratory of Regenerative Medicine, The First Affiliated Hospital of Guangxi Medical University, Nanning, Guangxi 530021, China.; 2 Life Sciences Institute, Guangxi Medical University, Nanning, Guangxi 530021, China.; 3 Department of Orthopaedics Trauma and Hand Surgery, The First Affiliated Hospital of Guangxi Medical University, Nanning, Guangxi 530021, China.; 4 Genetic and Metabolic Central Laboratory, Guangxi Birth Defects Research and Prevention Institute, Maternal and Child Health Hospital of Guangxi Zhuang Autonomous Region, Nanning, Guangxi 530021, China.

**Keywords:** biomimetic ECM collagen hydrogel, stem cell niches, POU5F1, AMPK/mTOR signaling pathway, autophagy

## Abstract

**Background:**

As a main component of the tissue microenvironment, the extracellular matrix (ECM) provides an instructive niche that regulates stem cell differentiation. Biomimetic ECM collagen hydrogels have been well regarded as ideal scaffold for cartilage tissue engineering due to their ability to promote chondrogenic differentiation of bone marrow mesenchymal stem cells (BMSCs). However, the molecular mechanisms underlying this inductive effect remain incompletely elucidated.

**Methods:**

In the present study, mRNA microarray analysis was performed to investigate the molecular mechanisms involved in collagen hydrogel-mediated chondrogenic induction. Real-time quantitative polymerase chain reaction (RT-qPCR), western blot, immunofluorescence analyses, etc. were used to validate the relevant pathways.

**Results:**

The results demonstrated that POU class 5 homeobox 1 (POU5F1), a transcription factor associated with stem cell differentiation and autophagy, was highly expressed in cells induced by collagen hydrogel. Differentially expressed genes (DEGs) were predominantly enriched in the AMP-activated protein kinase (AMPK) / mammalian target of rapamycin (mTOR) signaling pathway. Knockdown of POU5F1 suppressed activation of the AMPK/mTOR pathway and subsequently reduced intracellular autophagic flux, leading to impaired cartilage regeneration. These effects were partially reversed by treatment with rapamycin (RaPa), an mTOR inhibitor.

**Conclusions:**

The findings highlight a critical role of autophagy in chondrogenic induction mediated by biomimetic ECM collagen hydrogel and provide mechanistic insight that may inform the rational design optimization of cartilage repair biomaterials.

## Introduction

Articular cartilage exhibits limited capacity for self-repair due to the absence of blood and lymphatic vessels. Consequently, cartilage damage is difficult to restore and often progresses to osteoarthritis. Bone marrow mesenchymal stem cells (BMSCs) represent a population of progenitor cell with multipotent differentiation capacity and are therefore considered ideal seed cells for cartilage regeneration 1. Stem cells reside within a three-dimensional (3D) niche microenvironment that provide instructive cues, including soluble factors and cell-matrix interactions, which are essential for chondrogenic differentiation. Although the use of soluble growth factors has been widely adopted for stem cell induction, accumulation evidence suggests that stem cell culture within an appropriate extracellular matrix (ECM) can also promote differentiation. For instance, stem cells cultured in decellularized matrix have been shown to recapitulate functional specialization and morphogenesis of differentiated cell types with greater speed and efficiency than conventional growth factor-driven approaches 2. These findings indicated that the ECM functions as a holistic instructive niche that integrates multiple signals to regulate stem cell fate.

ECM-mediated regulation of stem cell reprogramming involves coordinated signaling changes across multiple subcellular levels, ranging from the cell-ECM interface at the plasma membrane to the cytoskeleton and intracellular organelles 3-5. Previous studies have shown that ECM-associated proteins regulating cell phenotype differentiation can influence lysosomes functions and initiate autophagy-dependent metabolic process. Lysosomes receive and degrade intracellular components through autophagosomes, thereby driving metabolic reprogramming and providing signals for stem cell lineage specification 6. ECM-regulated autophagy plays an essential role in tissue repair by guiding cellular functions during regeneration 7. Disruption of autophagy in stem cells leads to mitochondria accumulation compromises regenerative capacity 8. Impaired autophagosome-lysosome fusion, resulting in defective autophagosome degradation, has also been observed in the undifferentiated mesenchymal stem cell (MSCs) 9. These accumulated autophagosomes are rapidly cleared upon activation of autophagy under differentiation condition. Meanwhile, autophagy is induced during postnatal development and facilitates the secretion of type II collagen during chondrogenesis 10. Inhibition of autophagy by reducing the conjugation of ATG12-ATG5 leads to achondroplasia 11. Collectively, these reports revealed that ECM-mediated lysosomal autophagy can supply cells with anabolic precursors and energy required to support metabolic, structural and morphology remodeling requested by chondrogenic differentiation of stem cells. However, the mechanisms by which ECM mediates autophagy to regulate chondrogenic differentiation remain insufficiently defined.

POU class 5 homeobox 1 (POU5F1), also known as Oct3/4, is a member of the octamer-binding transcription factor family. As a regulator of autophagy, it is a key regulatory factor for the pluripotency control and chondrogenic fate acquirement of embryonic stem cells (ESCs) 12-14. Overexpression of POU5F1 promoted ESCs from spontaneously differentiating into trophectoderm and towards endoderm and mesoderm 15. Given that MSCs involved in cartilage formation primarily originated from the mesoderm, POU5F1 may have regulatory potential in directing MSC differentiation toward a chondrogenic lineage. Liu *et al.* found that overexpression of POU5F1 significantly increased the mRNA expression level of the chondrocyte marker *Col2a1* and enhanced the production of type II collagen and cartilage-specific proteoglycans in MSCs microsphere culture experiments 16. It was also found that silencing of POU5F1 inhibited MSC proliferation and chondrogenic differentiation 17, whereas overexpression of POU5F1 preserved MSCs characteristics 18. In addition, collagen-based hydrogel had been reported to provide a functional stem cell niche that enhances POU5F1 expression during wound healing 19. Although POU5F1 is implicated in lineage differentiation of stem cell, autophagy regulation, and niche signaling, the involvement of POU5F1 in ECM-mediated autophagy during chondrogenesis remains insufficiently understood.

We have previously developed a biomimetic ECM collagen hydrogel with cartilage inductive capacity. It can induce chondrogenic differentiation of BMSCs in the absence of any additional growth factors, which can be served as an ideal scaffold for tissue engineered cartilage construction 20, 21. In the present study, a tissue-engineered cartilage model based on collagen hydrogel was established, and mRNA microarray analysis was employed to elucidate the molecular mechanisms underlying collagen hydrogel-induced chondrogenic differentiation of BMSCs. These findings revealed that collagen hydrogel induced cartilage differentiation of BMSCs was closely associated with autophagy-related signaling pathways including lysosome and mammalian target of rapamycin (mTOR) pathways. Protein-protein interaction analysis confirmed that POU5F1 was a potential key regulator linking collagen hydrogel stimulation to autophagy activation during chondrogenic differentiation. In general, the results indicated that upregulation of POU5F1 activated autophagy through the AMPK-activated protein kinase (AMPK)/mTOR signaling pathway, representing a principal mechanism underlying collagen hydrogel-induced cartilage formation. These findings establish the connection between biomimetic ECM materials and molecular regulatory networks, providing an overall picture for the optimization design of clinical cartilage tissue engineering scaffolds.

## Results

### Intra-hydrogel culture promoted the viability of BMSCs for chondrogenic differentiation

As shown in **Figure S1**, the ECM biomimetic collagen hydrogel exhibited a porous architecture (**Figure S1A**), along with favorable degradability (**Figure S1B**), swelling capacity (**Figure S1C**) and gelation properties (**Figure S1D**). The results of live-dead cell staining were presented in **Figure S2A**, demonstrating a gradual increase of viable cells with prolonged culture duration, while only a small population of dead cells was observed. Cells could be evenly distributed and proliferate normally within the hydrogel. Cells stained with microfilament red fluorescent probe were used to observe the cytoskeleton. As shown in **Figure S2B**, cells displayed a rounded morphology with limited cytoskeletal extension on day 0, whereas the formation of polygonal actin filament structures increased progressively with culture time. In accordance with the result of F-actin staining, cartilage-like structures that stained by HE staining appeared at day 7, 14 and 21 (**Figure S3A**). In the 3D *in vitro* culture model, toluidine blue staining intensity gradually increased over time, indicating progressive ECM deposition (**Figure S3B**). Immunofluorescence analysis also showed a gradual increase in COL2A1 and SOX9 protein levels in cells cultured within collagen hydrogel compared to 0d (*p <* 0.001) (**Figure S3C**-**F**). In contrast, expression levels of fibrotic (COL I) and hypertrophic (COL X) chondrocyte markers did not change significantly among the four groups (**Figure S4**). These findings suggested that intra-collagen hydrogel culture effectively supported cell viability and promoted the chondrogenic differentiation of BMSCs.

### Identification of signaling pathways and key gene involved in collagen hydrogel-induced chondrogenesis

**Figure 1:**
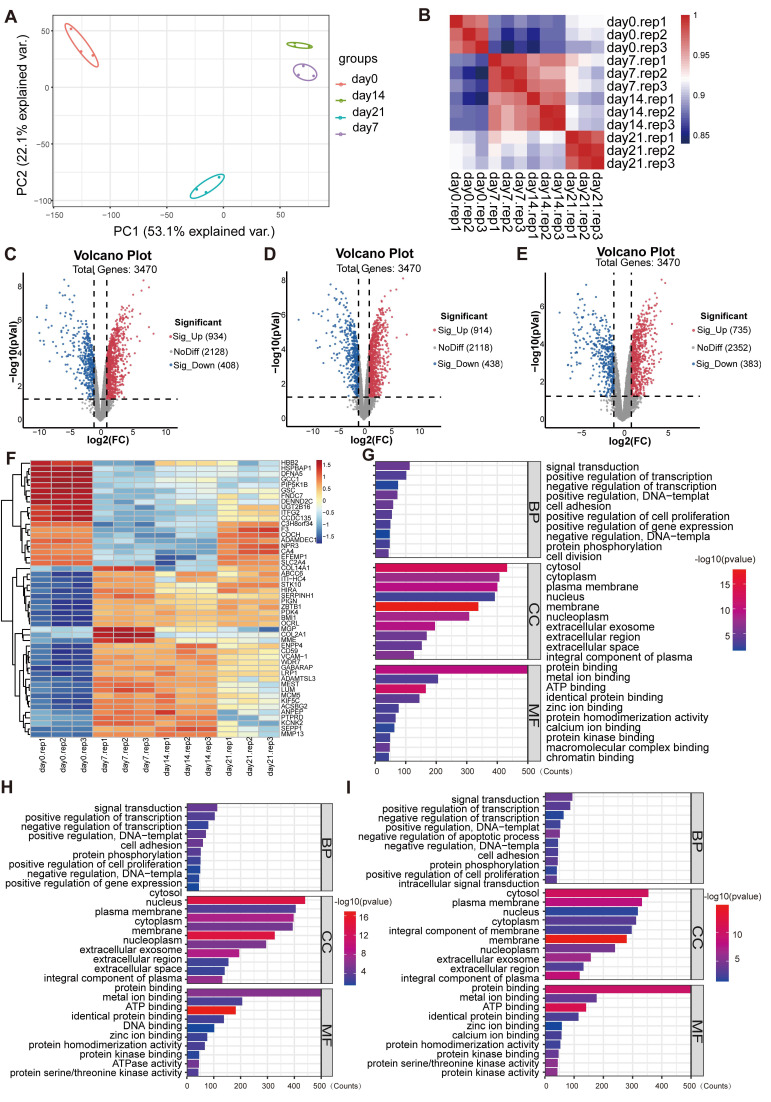
** Gene expression profiling analysis of DEGs in BMSCs induced by collagen hydrogel for 7, 14, and 21 days.** (**A**) PCA of global gene expression profiles. (**B**) Pearson correlation analysis of expression profile data. (**C-E**) Volcano plots of DEGs in day 7 (**C**), day 14 (**D**), and day 21 (**E**) (Red dots indicate significantly upregulated genes and blue dots indicate significantly downregulated genes). (**F**) Heatmap of the top 50 DEGs with the smallest p-values. (**G-I**) GO enrich analysis of the DEGs at day 7 (**G**), day 14 (**H**), and day 21 (**I**).

Principal component analysis (PCA) and Pearson correlation analysis were performed to assess the overall differences in the expression profiles among the experimental groups. The PCA results showed that gene expression in the cells of each group was quite different during the three-dimensional incubation (**Figure 1A**). Compared with day 0, the gene expression trends of the principal components were altered on day 7, 14 and 21, where the gene expression profile was similar between 7 and 14 days. In addition, the Pearson correlation analysis results showed that the three replicate samples in each group were highly associated and reproducible, whereas gene expression trends were similar on day 7 and 14 and different from day 21 (**Figure 1B**). As shown in the **Figure 1C**-**E**, there were 3470 valid genes in each group induction for days 7, 14 and 21 relative to day 0. Among these, 934, 914, and 735 genes were significantly upregulated at days 7, 14, and 21, respectively, whereas 408, 438, and 383 genes were significantly downregulated at the corresponding time points. Differentially expressed genes (DEGs) that expressed in the three induction groups simultaneously were analyzed by clustering (**Figure 1F**). It demonstrated that the gene expression trends of day 0 and other groups differed significantly.

**Figure 2:**
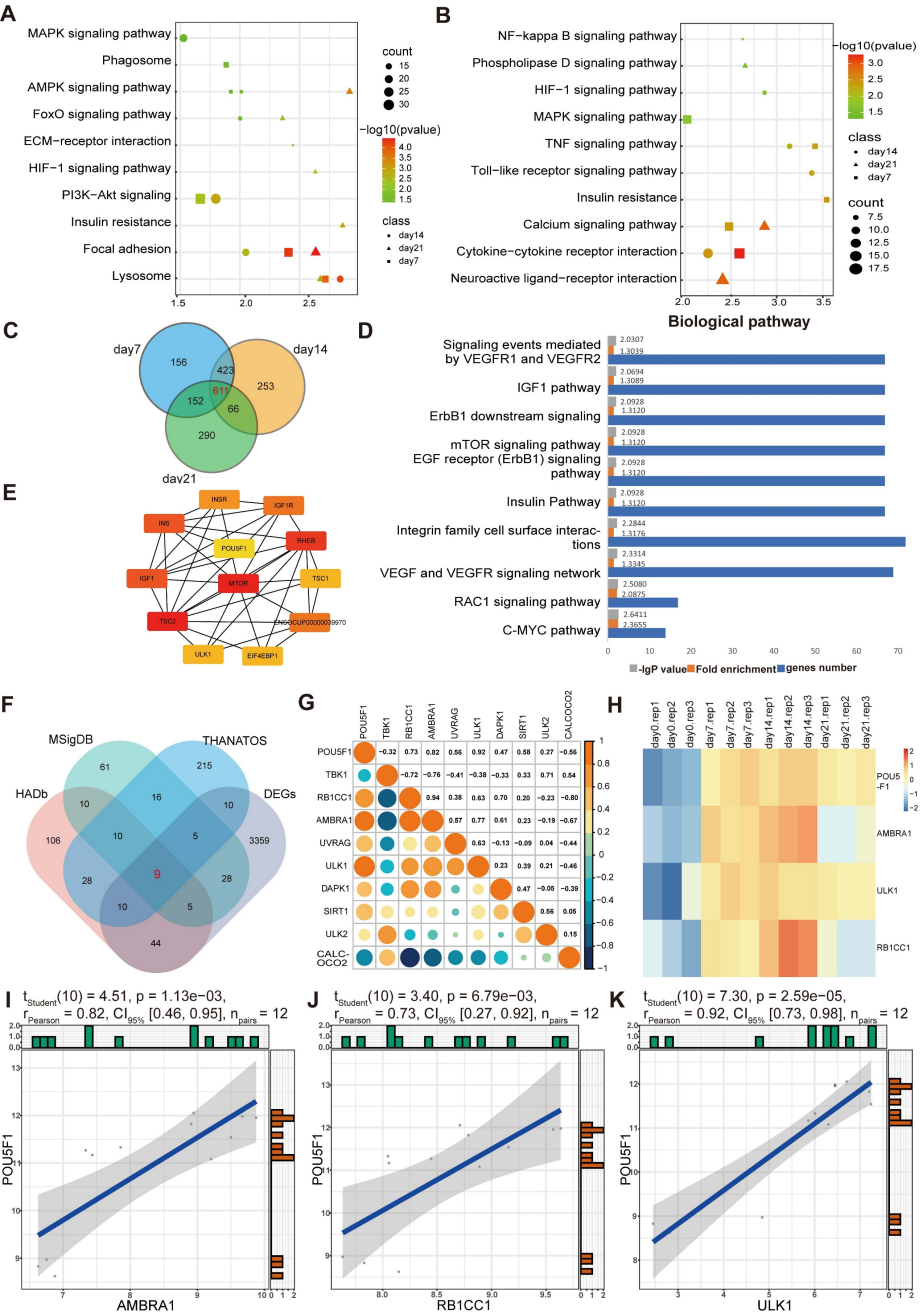
** Functional enrichment analysis of DEGs in BMSCs induced by collagen hydrogel for 7, 14 and 21 days.** (**A** and **B**) KEGG pathway enrichment analysis of up-DEGs (**A**) and down-DEGs (**B**) in 7, 14 and 21 days. (**C**) Venn diagram showed the intersection of DEGs among days 7, 14, and 21 groups. (**D**) Pathway enrichment analysis of the 611 intersecting DEGs identified in (**C**). (**E**) Protein-protein interaction network of genes enriched in AMPK/mTOR signaling pathway. (**F**) Venn diagram illustrated the intersection between DEGs and ARGs. (**G**) Pearson correlation analysis between POU5F1 and ARGs. (**H**) Heatmap showed expression profiles of POU5F1 and ARGs at different culture time points. (**I-K**) Scatter plots depicted correlations between POU5F1 and AMBRA1 (**I**), RB1CC1 (**J**) and ULK1 (**K**).

The biological functions enriched among the DEGs were examined using Gene Ontology (GO) analysis. It demonstrated that, within the biological process (BP) category, DEGs were mainly involved in signaling transduction, positive regulation of transcription, protein phosphorylation, cell adhesion, and cell proliferation (**Figure 1G**-**I**). In molecular function (MF) category, DEGs were mainly related to protein, protein kinase and metal ion bindings. In the cellular component (CC) category, they were distributed across multiple cellular compartments, including cytoplasm, nucleus, cell membrane and extracellular part. Signaling pathways associated with these DEGs were further screened using Kyoto Encyclopedia of Genes and Genomes (KEGG) enrichment analysis. As presented in the **Figure 2A** and **2B**, the enrichment of up-regulated DEGs were mainly concentrated in the AMPK signaling pathway, focal adhesion, lysosome pathway and so on, while the down-regulated DEGs were mainly enriched in the cytokine-cytokine receptor interaction, calcium and TNF signaling pathway, among others. To identify the signaling pathways that affect the cartilage formation of BMSCs sustainably, the DEGs of 7, 14, and 21 days were intersected and a total of 611 common DEGs were screened (**Figure 2C**). Then the pathway enrichment analysis of these 611 genes was carried out using FunRich software, and they were mainly enriched in RAC1, C-MYC and mTOR signaling pathways (**Figure 2D**). It has been reported that AMPK/mTOR signaling pathway is involved in the mesoderm differentiation of embryos, which is the origin of the stem cells for chondrogenesis 22. Thus, the protein-protein interaction analysis between the genes that enriched in AMPK/mTOR signaling pathway was performed and the result indicated that POU5F1 was the key gene for collagen hydrogel-induced chondrogenesis (**Figure 2E**).

Subsequently, nine common genes were obtained by interacting autophagy-related genes (ARGs) and DEGs (**Figure 2F**). The correlation between POU5F1 and autophagy-associated signaling was determined using Pearson correlation analysis, which revealed negative correlations with two genes and positive correlations with seven genes (**Figure 2G**). For further visualization, heatmaps (**Figure 2H**) and scatter plots (**Figure 2I**-**K**) were generated for the three genes with the highest correlation coefficients. The results showed that POU5F1, AMBRA1, ULK1 and RB1CC1 were expressed at relatively higher levels at 7, 14 and 21days compared to 0d, and expression trends of POU5F1 were positively correlated with those of key ARGs. These suggested that POU5F1 may activate autophagy by mediating AMPK/mTOR signaling pathway during collagen hydrogel-induced chondrogenic differentiation of BMSCs.

### Expression of POU5F1 and autophagy-related markers in BMSCs induced by collagen hydrogel

**Figure 3:**
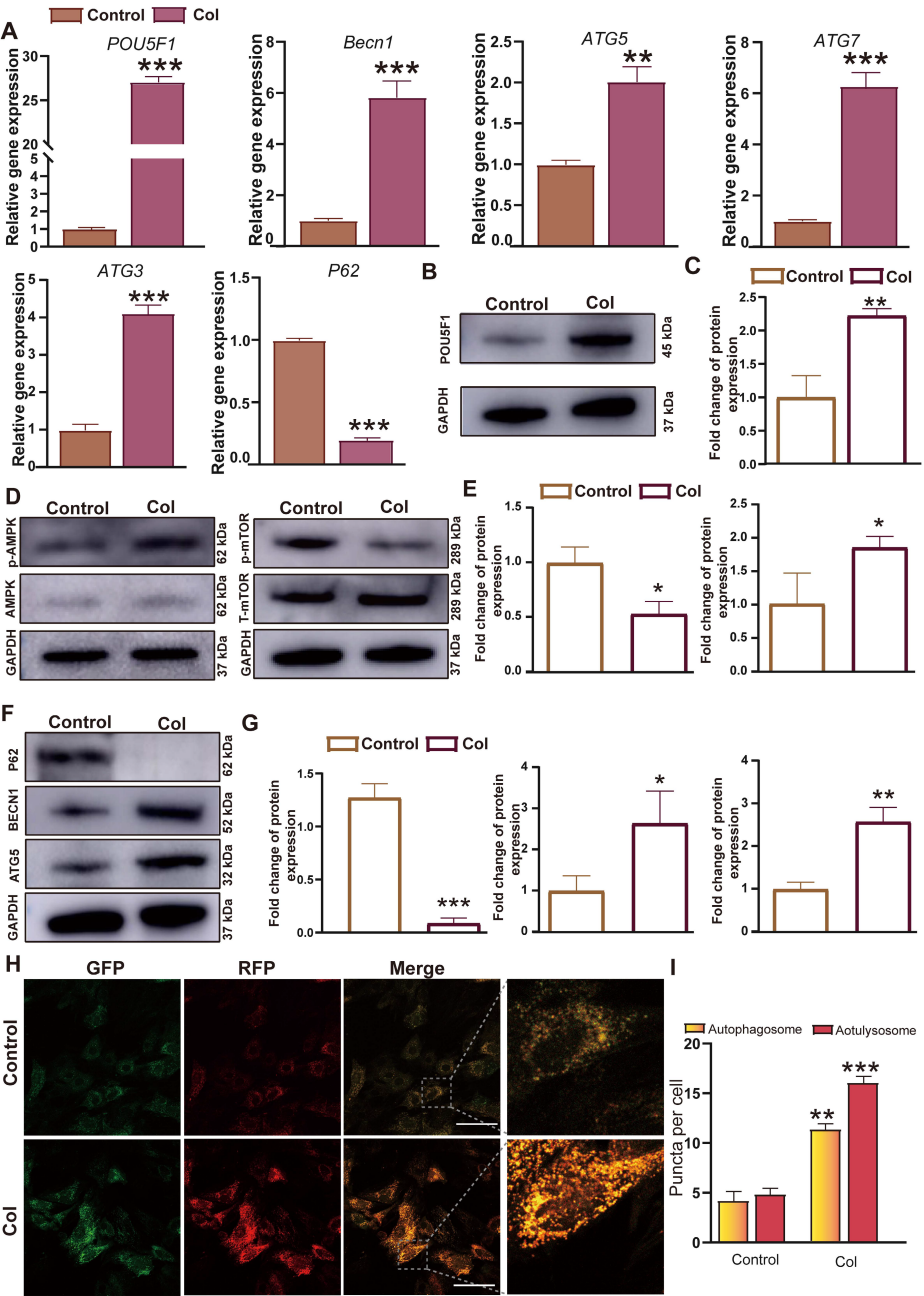
** Intra-collagen hydrogel culture promoted expression of POU5F1 and autophagy-related markers in BMSCs.** (**A**) Relative mRNA expression levels of *POU5F1*, *Becn1*, *ATG5*, *ATG7*, *ATG3* and *P62*. (**B**) Protein expression level of POU5F1. (**C**) Semi-quantification analysis of protein level shown in (**B**). (**D**) Protein expression levels of phosphorylated AMPK (p-AMPK) and phosphorylated mTOR (p-mTOR). (**E**) Semi-quantification analysis of protein expression shown in (**D**). (**F**) Protein expression levels of P62, BECN1 and ATG5. (**G**) Semi-quantification analysis of protein expression shown in (**F**). (**H**) Autophagic flux in BMSCs induced by collagen hydrogel. (**I**) Semi-quantification analysis of autophagosomes and autolysosomes in (**H**) (Scale bar: 50 μm; Mean ± SD, n = 3; *p < 0.05, **p < 0.01, ***p < 0.001).

Since the gene expression profiling analysis indicated that activation of autophagy through the AMPK/mTOR signaling pathway mediated by POU5F1 may represent a key mechanism underlying collagen hydrogel-induced chondrogenesis, real-time quantitative polymerase chain reaction (RT-qPCR), Western blot and autophagic flux analyses were performed to further confirm their expression levels in BMSCs culture within collagen hydrogel or under control conditions for 7 days. It showed that mRNA expression levels of *POU5F1* and ARGs including *Becn1*, *ATG5*, *ATG7*, and *ATG3* were significantly higher in the collagen hydrogel (Col) group compared to the control group, whereas expression of autophagy substrate *P62* was reduced in the Col group (*p <* 0.01) (**Figure 3A**). These findings were consistent with the gene expression profiling data. Meanwhile, protein expression levels of POU5F1, BECN1, ATG5, and phosphorylated AMPK were significantly increased in the Col group relative to the control group (*p <* 0.05) (**Figure 3B**-**G**), while expression of P62 and phosphorylated mTOR were suppressed. After 7 days of induction, autophagic flux was visualized using confocal laser scanning microscopy (CSLM). Compared with the control group, a significant increase in both autophagosomes (yellow puncta) and autolysosomes (red puncta) was observed in the Col group (**Figure 3H** and **I**). The results reflected that activation of POU5F1, modulation of AMPK/mTOR signaling pathway, and enhanced autophagic activity are involved in collagen hydrogel-induced chondrogenic differentiation of BMSCs.

### POU5F1 activated autophagy by mediating AMPK/mTOR signaling during collagen hydrogel-induced chondrogenesis

**Figure 4:**
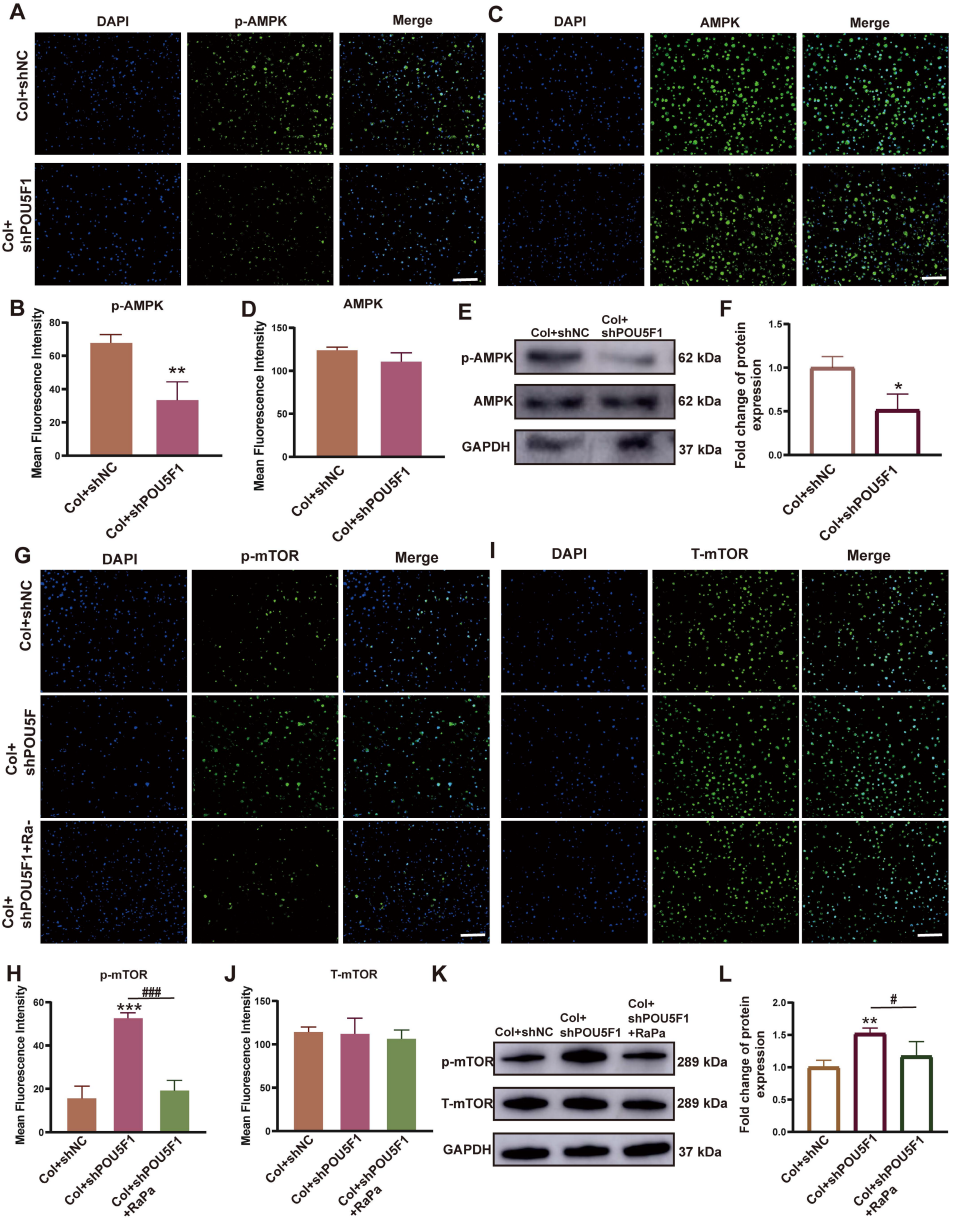
** Knockdown of POU5F1 inhibited the AMPK/mTOR signaling pathway in BMSCs induced by collagen hydrogel.** (**A**) Immunofluorescence staining for p-AMPK. (**B**) Semi-quantification analysis of p-AMPK shown in (**A**). (**C**) Immunofluorescence staining for AMPK. (**D**) Semi-quantification analysis of AMPK shown in (**C**). (**E**) Western blot analysis detected protein levels of AMPK and p-AMPK. (**F**) Semi-quantification analysis of p-AMPK/AMPK shown in (**E**). (**G**) Immunofluorescence staining for p-mTOR. (**H**) Semi-quantification analysis of p-mTOR shown in (**G**). (**I**) Immunofluorescence staining for T-mTOR. (**J**) Semi-quantification analysis of T-mTOR shown in (**I**). (**K**) Western blot analysis detected protein levels of p-mTOR and T-mTOR. (**L**) Semi-quantification analysis of p-mTOR/T-mTOR shown in (**K**) (Scale bars: 200 μm; Mean ± SD, n = 3; *p < 0.05, **p < 0.01, ***p < 0.001 *vs.* Col+shNC; ^#^*p <* 0.05, ^###^*p <* 0.01 for intergroup comparisons).

To investigate the role of POU5F1 in regulating the AMPK/mTOR signaling pathway and autophagy during collagen hydrogel-induced chondrogenic differentiation of BMSCs, a stable POU5F1 knockdown BMSC cell line was established. Both mRNA and protein expression levels of POU5F1 were reduced in shPOU5F1 group compared with Col+shNC group, regardless of intra-collagen hydrogel culture conditions (*p <* 0.01), indicating effective silencing of POU5F1 in the BMSCs (**Figure S5**). Using POU5F1-deficient cells, the regulatory role of POU5F1 in the AMPK/mTOR signaling pathway was further examined. At 7 days of induction, the results of immunofluorescence staining showed knockdown of POU5F1 significantly inhibited the phosphorylation of AMPK (p-AMPK) compared with the Col+shNC group, while expression of AMPK remained unchanged (**Figure 4A-D**). Western blot analysis also further confirmed suppression of AMPK activation, with the p-AMPK/AMPK ratio decreased by 51.7% in Col+shPOU5F1 group compared with Col+shNC group (**Figure 4E** and **F**). In addition, the phosphorylation of mTOR (p-mTOR) was increased in the Col+shPOU5F1 group compared to the Col+shNC group, whereas this effect was attenuated following treatment with rapamycin (RaPa), an mTOR inhibitor (**Figure 4G** and** H**). Total mTOR (T-mTOR) protein levels were similar among all the three groups examined (**Figure 4I** and **J**). Quantitative analysis showed that the p-mTOR/mTOR ratio was elevated by 47.9% in the Col+shPOU5F1 group compared with Col+shNC group (**Figure 4K** and **L**). And there was no obvious difference between Col+shNC and Col+shPOU5F1+RaPa groups. These findings suggested that knockdown of POU5F1 affected the AMPK/mTOR signaling pathway during collagen hydrogel-induced chondrogenic differentiation of BMSCs.

**Figure 5:**
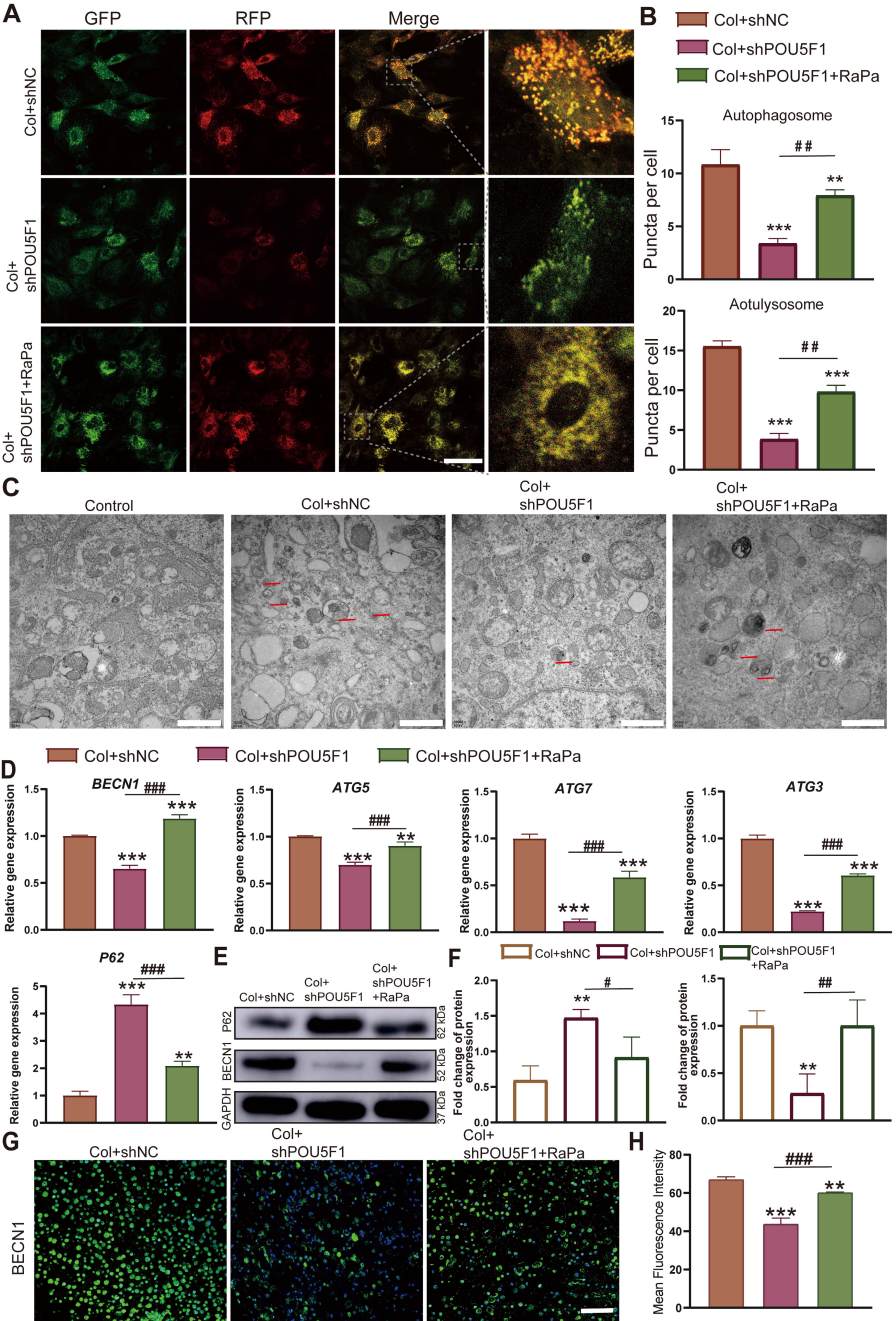
** POU5F1 regulated autophagy in BMSCs induced by collagen hydrogel.** (**A**) Autophagic flux in mRFP-GFP-LC3-transfected BMSCs. Autophagosomes are labeled by red and green fluorescence (yellow puncta), autolysosomes are labeled by red fluorescence (red puncta) (Scale bar: 50 μm). (**B**) Quantification of fluorescent puncta shown in (**A**). (**C**) Transmission electron microscopy images of BMSCs cultured with/without collagen hydrogel (Scale bars: 1 μm). (**D**) Relative mRNA expression levels of *POU5F1*, *Becn1*, *ATG5*, *ATG7*, *ATG3* and *P62* in the shNC-, shPOU5F1-transfected or RaPa-treated BMSCs cultured within collagen hydrogel. (**E**) Protein expression of P62 and BECN1 detected by Western blot. (**F**) Semi-quantification analysis of P62 and BECN1 expression shown in (**E**). (**G**) Protein expression of BECN1 detected by immunofluorescence staining (Scale bar: 200 μm). (**H**) Semi-quantification analysis of BECN1 expression shown in (**G**) (Mean ± SD, n = 3; **p < 0.01, ***p < 0.001 *vs.* Col+shNC; ^#^*p <* 0.05, ^##^*p <* 0.01, ^###^*p <* 0.01 for intergroup comparisons).

To further assess the role of POU5F1 in autophagy activation through modulation of the AMPK/mTOR signaling pathway during collagen hydrogel-induced chondrogenesis, an mRFP-GFP-LC3 adenoviral reporter system was applied in POU5F1-knockdown BMSCs. The result of autophagic flux showed that the POU5F1-knockdown cells significantly reduced the formation of autophagosomes (yellow puncta) and autolysosomes (red puncta) compared with the Col+shNC group, while inhibition of mTOR by RaPa restored the formation of autophagosomes and autolysosomes (**Figure 5A** and** B**). Transmission electron microscopy (TEM) further confirmed a significant reduction in autophagosome accumulation in the Col+shPOU5F1 group relative to the Col+shNC group (**Figure 5C**). The accumulation of autophagosomes could be restored in some degree by activating autophagy using the mTOR inhibitor. Meanwhile, mRNA expression levels of autophagy-related markers, including *BECN1*, *ATG5*, *ATG7* and *ATG3* were decreased 65.6%, 69.8%, 12.4% and 22.5% in POU5F1-knockdown BMSCs (*p <* 0.001), whereas expression of autophagic substrate *P62* was correspondingly increased 230.7% (**Figure 5D**). Similarly, protein expression of BECN1 that detected by Western blot analysis (**Figure 5E** and** F**) and immunofluorescence staining (**Figure 5G** and** H**) was suppressed following POU5F1 knockdown (*p <* 0.01), accompanied by corresponding accumulation of p62 protein. Alterations in the autophagy-related genes and protein expression that affected by knockdown of POU5F1 all could be reversed by treatment with autophagic activator (RaPa) (*p <* 0.01). Collectively, these findings indicated POU5F1 could activate autophagy by mediating AMPK/mTOR signaling pathway in BMSCs cultured within collagen hydrogel.

### POU5F1 activated autophagy to promote chondrogenesis of BMSCs cultured in collagen hydrogel

**Figure 6:**
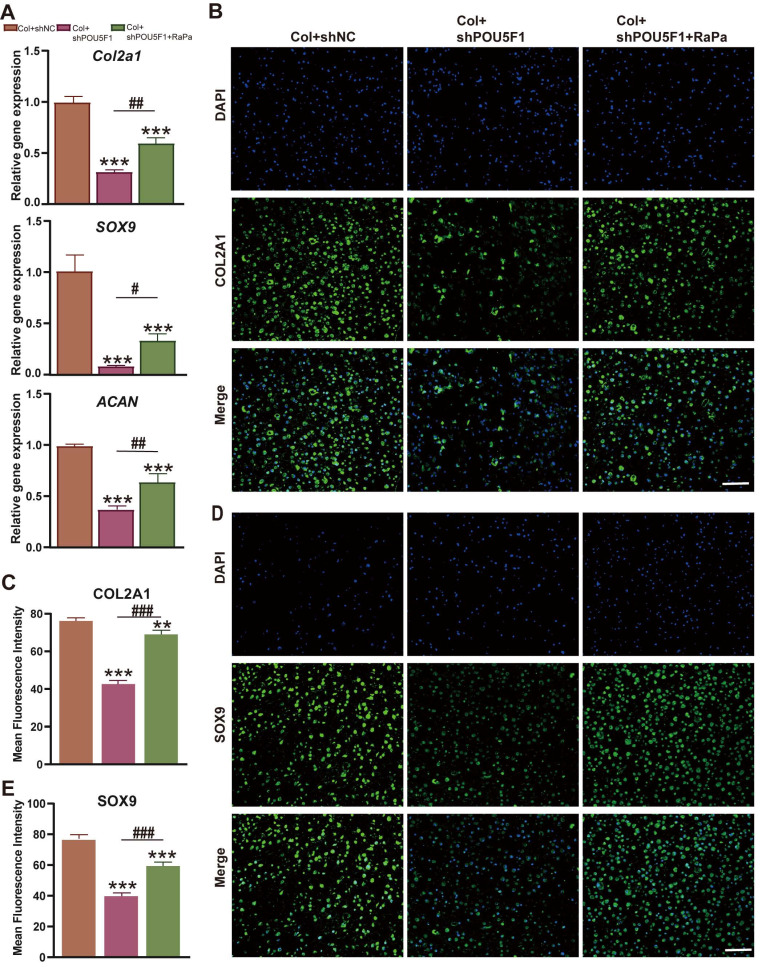
** Effects of POU5F1 and autophagy on chondrogenesis of BMSCs induced by collagen hydrogel.** (**A**) Relative mRNA expression levels of *Col2a1*,* SOX9* and *ACAN*. (**B**) Immunofluorescence staining of COL2A1. (**C**) Semi-quantification analysis of protein expression shown in (**B**). (**D**) Immunofluorescence staining of SOX9. (**E**) Semi-quantification analysis of protein expression shown in (**D**) (Scale bars: 200 μm; Mean ± SD, n = 3; **p < 0.01, ***p < 0.001 *vs.* Col+shNC; ^#^*p <* 0.05, ^##^*p <* 0.01, ^###^*p <* 0.01 for intergroup comparisons).

To explore the role of POU5F1 in modulating autophagy during chondrogenic differentiation of BMSCs cultured within collagen hydrogel, expression levels of cartilage-related markers were examined using RT-qPCR and immunofluorescence staining. The results demonstrated that cartilage specific genes, including *Col2a1*,* SOX9*, and *ACAN*, were obviously downregulated in the Col+shPOU5F1 group compared with the Col+shNC group (*p <* 0.001) (**Figure 6A**). However, treatment with RaPa resulted in partial restoration of these gene expression levels (*p <* 0.05). Consistently, knockdown of POU5F1 led to marked reductions in COL2A1 and SOX9 protein expression (**Figure 6B**-**E**). Restoration of autophagic activity through mTOR inhibition significantly rescued the production of these chondrogenic proteins in shPOU5F1 cells (*p <* 0.001). Collectively, POU5F1 played a crucial role in promoting intracellular autophagy to drive collagen hydrogel-induced chondrogenic differentiation of BMSCs by mediating AMPK/mTOR signaling pathway.

### POU5F1 promoted cartilage defect repair by regulating autophagy

**Figure 7:**
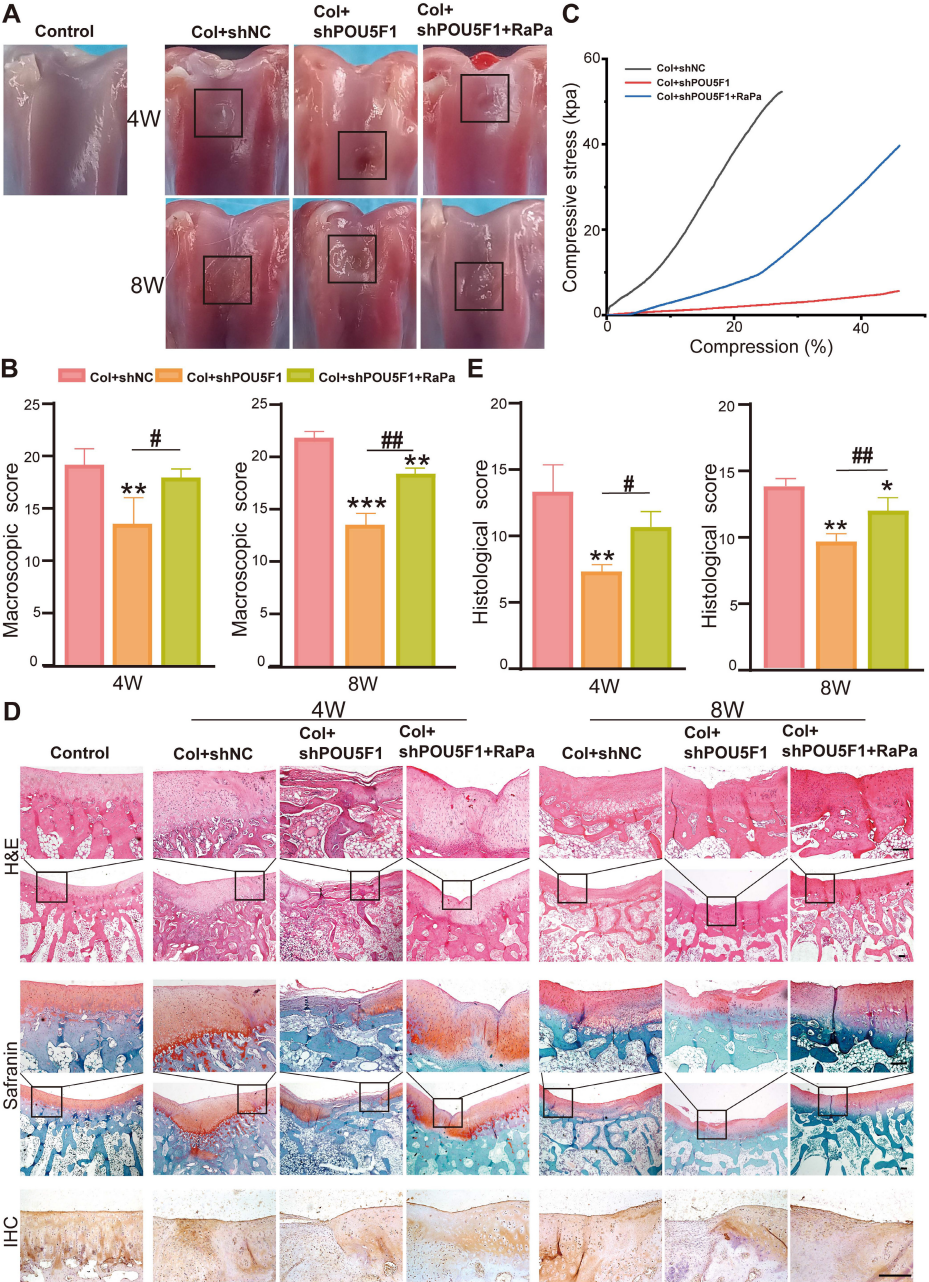
**POU5F1 enhanced collagen hydrogel-mediated cartilage defect repair *in vivo***. (**A** and **B**) Macroscopic observation and scores of cartilage defects after 4 weeks and 8 weeks of repair. (**C**) Biomechanical testing of the engineered cartilage. (**D**) Histological evaluation of repaired cartilage at 4 and 8 weeks using HE, Safranin O/fast green staining and immunohistochemical staining for COL2A1. (**E**) Histological scores of cartilage following implantation of the cellular collagen hydrogel scaffolds into the defect sites (Scale bars: 200 μm; Mean ± SD, n = 5; *p < 0.05, **p < 0.01, ***p < 0.001 *vs.* Col+shNC; ^#^*p <* 0.05, ^##^*p <* 0.01 for intergroup comparisons).

A rabbit knee cartilage defect model was established to explore the roles of POU5F1 and autophagy in collagen hydrogel-mediated cartilage regeneration. BMSCs transfected with shNC or shPOU5F1 loaded with scaffolds were implanted into the defect site. Macroscopic observation and score of articular cartilage repair were performed after 4 and 8 weeks of treatment. The results showed that the articular cartilage defects in all groups were filled with newly formed tissue to varying extents (**Figure 7A**). After 4 weeks, cartilage surface of the defect sites in the Col+shNC group were largely filled with engineered cartilage, but the boundary between the edge of normal cartilage and neo-tissue was still obvious. The regenerated tissue was partially filled in the Col+shPOU5F1 group. Compared with the Col+shPOU5F1 group, cartilage repair was significantly improved in the Col+shPOU5F1+RaPa group. After 8 weeks, new cartilage that appeared close to the normal cartilage in the Col+shNC group was completely filled the defects and well fused with the in-situ tissue. Residual defects remained evident in the Col+shPOU5F1 group. Even though the surface of new cartilage in the Col+shPOU5F1+RaPa group remained rough, significant improvement in fusion was shown compared with the Col+shPOU5F1 group. Consistent with macroscopic observations, macroscopic scores (4 weeks: 19.3±1.2; 8 weeks: 22±0.8) were the highest in the Col+shNC group compared with Col+shPOU5F1 group (4 weeks: 13.7±2.1; 8 weeks: 13.7±0.9) and Col+shPOU5F1+RaPa group (4 weeks: 18±0.8; 8 weeks: 18.3±0.5) (**Figure 7B**). In agreement with macroscopic evaluation, biomechanical properties of engineered cartilage were decreased in the Col+shPOU5F1 group in comparison with Col+shNC group, whereas partial recovery of biomechanical performance was observed in the Col+shPOU5F1+RaPa group (**Figure 7C**).

The quality of repaired tissue within articular cartilage defects was further evaluated by HE, immunohistochemical and safranin O/fast green staining and histological scoring. At 4 weeks, better integration of repaired tissue with surrounding normal cartilage and more organized chondrocyte-like cells were observed in the Col+shNC group (**Figure 7D**). In the POU5F1 knockdown group, only a few cells and disordered neo-tissue were exhibited. The regenerated tissues and chondrocyte-like cells in the Col+shPOU5F1+RaPa group were greater than those in the Col+shPOU5F1 group, although cellular organization remained suboptimal. Deposition of sulfated glycosaminoglycans (sGAG) and COL2A1, which are main components of ECM of articular cartilage, were assessed by Safranin O/fast green and immunohistochemical staining. Compared with the Col+shNC group, knockdown of POU5F1 showed negative and uniform staining for sGAG and COL2A1, and partially positive staining was observed following RaPa treatment. At 8 weeks, continuous hyaline-like cartilage was repaired in the Col+shNC group, as evidenced by smooth surface, improved cell filling, intense sGAG and COL2A1 staining. In contrast, the Col+shPOU5F1 group displayed persistent demarcation between native cartilage and repaired tissue, accompanied by predominantly negative sGAG and COL2A1 staining in the neo-tissue. Little boundary and abundant sGAG and COL2A1 staining were observed in the Col+shPOU5F1+ RaPa group.

Histological scores of the regenerated cartilage increased progressively over time in all groups. Consistent with macroscopic evaluation, significantly higher histological scores were shown in the Col+shNC group with 81.8% and 41.4% higher than that in the Col+shPOU5F1 group at 4 and 8 weeks (**Figure 7E**). Enhancement of autophagic activity by RaPa significantly elevated histological scores to 45.5% and 24.1% compared with the Col+shPOU5F1 group at 4 and 8 weeks. Overall, reduced cellular filling and diminished ECM deposition following POU5F1 knockdown, along with partial restoration by RaPa treatment, indicated that POU5F1-mediated autophagy plays a critical role in cartilage regeneration *in vivo* following implantation of BMSCs collagen hydrogel scaffolds.

## Discussion

**Figure 8:**
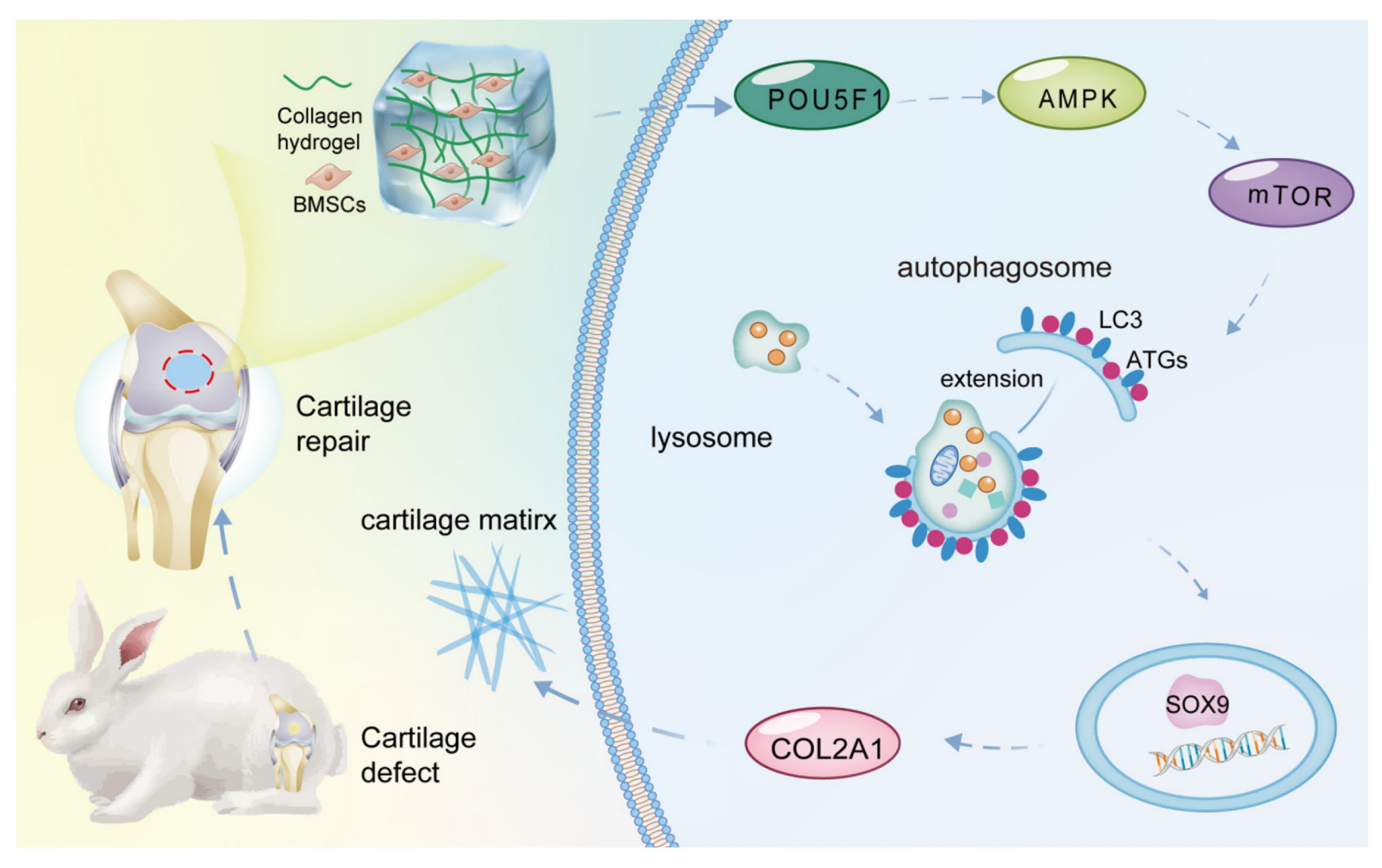
Schematic illustration of the molecular mechanism underlying collagen hydrogel-induced chondrogenic differentiation of BMSCs.

Collagen hydrogel, which mimics the cartilage ECM microenvironment for chondrogenic induction, can be used as a prime scaffold for cartilage tissue engineering 23. Through mRNA microarray analysis, the present study found that POU5F1 activates autophagy through the AMPK/mTOR pathway to promote chondrogenic differentiation of BMSCs cultured within collagen hydrogel (**Figure 8**). POU5F1-deficient BMSCs and autophagy activator RaPa used for function and mechanism studies further verify this regulatory mechanism both *in vitro* and *in vivo* models.

Autophagy has been proposed as a critical contributor to the therapeutic action of MSCs 9. It can affect functions of MSCs by modulating their activities and the autophagosomes recycle cellular components such as damaged organelles, lipids, and proteins via fusing with lysosomes for energy supplementation 24, 25. Consistent with the studies reported, enhanced viability of BMSCs cultured within collagen hydrogel was observed in the present study (**Figure S2A**). Correspondingly, autophagic activity and the expression of autophagy-related markers, including BECN1 and ATG5 were significantly enhanced following collagen hydrogel culture (**Figure 3F-I**). Moreover, DEGs in mRNA microarray were also mainly enriched in the autophagy signaling pathway like AMPK, lysosome and mTOR signaling pathways (**Figure 2A** and** D**,** Figure 3D-E**). As known to all that autophagy can be controlled by AMPK and mTOR signaling pathways, which regulate the activation and assembly of the ATG1/ULK1 complex 26. AMPK, a primary intracellular receptor of energy status, responds to energy depletion by modulating the ratio of p-mTOR/t-mTOR 27, 28. The mTORC1 complex interacts with ULK1/2, leading to phosphorylation of ULK1/2 and ATG13 under nutrient-sufficient conditions, thereby regulating the formation of autophagosomes 29. In addition, the formation of autophagosome membranes for fusion with lysosomes is mainly regulated by BECN1 30. These regulatory mechanisms collectively indicate that autophagy is an essential biological process underlying chondrogenic induction mediated by biomimetic ECM collagen hydrogel.

We found that POU5F1 was the key activator of autophagy during the chondrogenic induction of BMSCs induced by collagen hydrogel (**Figure 2E**). Knockdown of POU5F1 decreased the levels of autophagy (**Figure 5**) and chondrogenic markers (**Figure 6**). Consistently,* in vivo* cartilage repair showed that the engineered cartilage was also affected by knockdown of POU5F1 (**Figure 7**). However, all these impacts can be reversed by treatment with RaPa. In fact, POU5F1 has been reported as an essential regulator for autophagy activation and chondrogenesis 31, 32, which was in agreement with our study. The pluripotency-regulating effects of POU5F1 were accompanied by contrasting levels of autophagy. Autophagy could be robustly induced by POU5F1 during the reprogramming of mouse fibroblasts to induced pluripotent stem cells 33 and POU5F1 knock-down inhibited the autophagy machinery 34. In addition, the molecular interaction network of POU5F1 connected to regulation and growth of stem cells 35. Precise regulation of POU5F1 expression is necessary to maintains the ESCs pluripotency 36. These evidences shed light on the underlying mechanism of autophagy regulation during collagen hydrogel-induced chondrogenesis, highlighting the crucial role of the POU5F1 in orchestrating autophagy induction.

In summary, the present study elucidated the mechanism underlying collagen hydrogel-mediated chondrogenic induction, wherein the collagen hydrogel activated autophagy leads to elevation of BMSCs functions. Expression of POU5F1 as well as intracellular autophagic flux were increased during chondrogenic induction of BMSCs. Knockdown of POU5F1 significantly suppressed the activation of the AMPK/mTOR pathway and subsequently reduced autophagy. Restoration of autophagic flux in POU5F1-deficient BMSCs was achieved through RaPa-mediated mTOR inhibition. These findings not only provide new insight into the connection between cartilage-inductive biomaterial scaffolds and molecular regulatory network of chondrogenesis, but also conducive to the optimal design of materials in clinical cartilage defect repair.

## Materials and Methods

### Characterization of collagen hydrogel

The microstructure of the collagen hydrogel was examined by scanning electron microscopy (SEM; ZEISS, Germany) after freeze-drying. Hydrogel samples (10×10 mm) were prepared, freeze-dried, and weighed to obtain initial weight (W₀). For *in vitro* degradation test, samples were immersed in PBS followed by vacuum-dried, and weighed at set time point (Wt). Mass retention rate = (Wt/W₀) × 100%. For swelling test, samples were immersed in PBS and weighted at time point t (W_t_). Repeat the above measurement until two consecutive measurements show negligible mass change. Swelling ratio = ((Wt-W₀)/W₀) × 100%. A rotational rheometer (Waters, US) was used to measure the rheological properties of collagen hydrogel. The parameters were set at a frequency of 1 Hz and a strain of 1% at 37℃. The storage modulus (G′) and loss modulus (G″) were recorded.

### Cell culture

Bone marrow cavities were harvested from New Zealand rabbits (5 days old) following euthanasia under anesthesia for isolation of BMSCs. Cells were cultured with alpha-modified Eagle's medium (α-MEM, Hyclone, USA) containing 1% penicillin-streptomycin (Solarbio, Beijing, China) and 10% fetal bovine serum (FBS; EVERY GREEN, Hangzhou, China) and cultured in an incubator at 37 °C with 5% CO_2_.

### Construction of a 3D *in vitro* culture model

Collagen solution (15 mg/mL, National Biomaterial Engineering Research Center, Chengdu, China) was neutralized with NaOH (1M) at 4 °C. Third-passage BMSCs were digested and mixed with the collagen hydrogel (1×10^7^ cell/100 μL). Subsequently, the cell-hydrogel mixtures were transferred to the incubator at 37 °C for 10 minutes to allow gelation. Finally, the mixtures were cultured in the complete medium (DMEM supplemented with 10% FBS) for chondrogenic induction.

### Cell viability assay

Cells within the 3D culture model were harvested at day 0, 7, 14, and 21, respectively. A live/dead staining kit (Invitrogen, USA) was utilized to assess the cell viability. Dye solution containing 1% propidium iodide (PI) and 1% Calcein-AM were prepared by diluting in PBS. Cell specimens were stained for 5 min away from light and imaged in three-dimensional mode using a laser scanning confocal microscope (Leica, Germany) with excitation wavelengths of 488nm and 561nm. Semi-quantification analysis of fluorescence intensity was performed by using ImageJ software.

### F-actin staining

The staining solution was obtained by diluting the Actin-Tracker Red-555 (Beyotime, Shanghai, China) with PBS (1:150). Then specimens were fixed by 4% paraformaldehyde for 10 min. Then samples were dyed with staining solution for 60 min away from light. Finally, cells were stained with 4, 6-diamino-2-phenyl indole (DAPI; Solarbio, Beijing, China) for 5 min and observed using a confocal microscope. Semi-quantification analysis of fluorescence intensity was performed by using ImageJ software.

### Gene expression profiling on microarrays

Total RNA of cells in 3D culture model for 0, 7, 14, and 21 days was extracted with a HiPure Total RNA Kit (Mgenbio, Shanghai, China) and quantified with a NanoDrop2000 spectrophotometer. RNA samples were amplified and reversed-transcribed into complementary DNA (cDNA). After labeling, cDNA samples were placed on a microarray chip to undergo the hybridization process, and then the chip was washed and scanned. Eventually, data was extracted by using Agilent Feature Extraction Software. Since each gene corresponds to multiple probes, we randomly removed duplicate entries and deleted some undefined genes to ensure that there were only three replicates of expression values for each gene in each group. The gene expression profiling on microarray had been uploaded to the GEO database of National Center for Biotechnology Information (NCBI) with an accession number of GSE315306. Differential gene expression among groups was quantified by calculating log_2_ fold changes in mRNA expression levels.

### Differential expression analysis and function enrichment analysis

Principal component analysis (PCA), Pearson correlation analysis, and differential expression analysis of microarray data were performed according to previously reported methods 37. DEGs of each group were imported into the DAVID online website (https://cn.string-db.org/) and selected OFFICAL-GENE-SAMPLE as the input data type to obtain the results of enrichment analysis. Bar plots and bubble charts were generated using an online visualization platform (https://www.bioinformatics.com.cn/) to visualize the results of enrichment analysis. To clarify the mechanism of continuous influence on the chondrogenic differentiation of BMSCs during the 3D culture process, DEGs in three-time points were intersected to obtain the common ones and further analyzed by FunRich software to clarify the biological pathways involved.

### Correlation analysis of POU5F1 and key signaling molecules

ARGs were screened by combination with THANATOS database (https://ngdc.cncb.ac.cn/databasecommons/database/id/4471), Human Autophagy Database (http://www.autophagy.lu/) and Molecular Signatures Database (http://www.autophagy.lu/). Pearson correlation analysis was performed using the Pearson function package between POU5F1 and ARGs, and the three genes with the highest correlation were selected to plot scatter plots.

### Construction of POU5F1 knockdown BMSCs cell line

To silence POU5F1 expression in BMSCs, lentiviral vector (hU6-MCS-CMV-Puromycin) containing the short-hairpin RNA (shRNA) specifically targeting POU5F1 was constructed (GeneChem, Shanghai, China). A negative sequence (shNC) was used as control. The sequences were as follow: POU5F1-RNAi (107464-12)-5'CTGCAGAAAGAACTCGAGCAA3', negative control RNAi-5' TTCTCCGAACGTGTCACGT3'. Lentiviral transduction was performed by adding HitransG A reagent and virus at a multiplicity of infection (MOI) of 10 when cell confluence reached approximately 50%. Cell passaging was performed at the end of infection and stable shPOU5F1 and shNC cell lines were screened by using puromycin (2 μg/mL, Maclin, Shanghai, China).

### Cell treatments

Cells were seeded into the collagen hydrogel as descript previously. The experiments were divided into five groups: (1) Control: BMSCs were cultured in cluster; (2) Col: BMSCs were cultured within collagen hydrogel; (3) Col+shNC: BMSCs transferred with shNC and cultured within collagen hydrogel; (4) Col+shPOU5F1: BMSCs transferred with shPOU5F1 and cultured within collagen hydrogel; (5) Col+shPOU5F1+RaPa: BMSCs transferred with shPOU5F1 and cultured within collagen hydrogel treated with rapamycin (200 nmol/L).

### Autophagy detection

Autophagic flux was assessed using a tandem fluorescent mRFP-GFP-LC3 adenoviral reporter (GeneChem, Shanghai, China) to transfect normal or POU5F1-knockdown BMSCs. Stable expression was selected using G418 (100 μg/mL). Cells were cultured for 7 days and scanned with a laser confocal microscope. The level of intracellular autophagy was quantified by calculating the number of fluorescent puncta using ImageJ software. Colocalized red and green fluorescence (yellow puncta) represented autophagosomes, whereas red-only fluorescence indicated autolysosomes. The intracellular autophagy was further detected by TEM. Samples from each group were collected and processed by fixation, gradient dehydration, immersion, embedding, polymerization, sectioning and staining. Ultrastructural visualization of autophagosomes were performed using an H-7650 TEM (Hitachi, Japan).

### RT-qPCR analysis

Samples were lysed and the total RNA was separated by a total RNA small volume extraction kit (Magen, Guangzhou, China) according to our previous study 38. cDNA was prepared by reverse transcription using the PrimeScript RT reagent Kit (Takara, Japan). Primer sequences used in the present study were summarized in **Table S1**. Quantitative PCR was performed using a LightCycler® 96 Instrument under the following conditions: initial denaturation at 95 °C, followed by 40 cycles of denaturation at 95 °C for 10 seconds and annealing/extension at 60 °C for 60 seconds. Gene expression between different groups was standardized by GAPDH, and 2^-ΔΔt^ method was performed to calculate the relative expression levels of genes.

### Western blot analysis

Cells were lysed with RIPA (Beyotime, Shanghai, China), and protease inhibitors and phosphatase inhibitors were used to prevent protein degradation. BCA kit (Beyotime, Shanghai, China) was utilized to determine the concentrations of total protein and followed by denaturation in boiling water in the addition of sample loading buffer. Primary antibodies used in the experiment were POU5F1 (1:1000, Proteintech, Wuhan, China), BECN1 (1:1000, Proteintech, Wuhan, China), ATG5 (1:1000, Proteintech, Wuhan, China), P62 (1:1000, Proteintech, Wuhan, China), p-mTOR (1:1000, Cell Signaling Technology, USA), p-AMPK (1:1000, Cell Signaling Technology, USA), mTOR (1:1000, Cell Signaling Technology, USA), AMPK (1:1000, Cell Signaling Technology, USA) and GAPDH (1:1000, Thermo Fisher Scientific, USA). Different concentrations of gels (10% for less than 180 kDa and 6% for more than 180 kDa) were prepared for electrophoresis, and 0.45 µm membranes were selected for membrane transfer. Following transfer, the membranes were immersed in blocking buffer (Sangon, Shanghai, China) for 60 min and then incubated with primary antibody at 4°C for 12 h. After removing the primary antibody, the membranes were washed three times with TBST and incubated with the secondary antibody (Sangon Biotech, Shanghai, China) for 1 h at room temperature. Finally, ECL (Beyotime, Shanghai, China) was added for development and the bands were captured with Amersham Imager 600 (GE, USA).

### Construction of articular cartilage defect model

Forty-two New Zealand White rabbits (male, 8 weeks old) were obtained from the Experimental Animal Center of Guangxi Medical University (Nanning, Guangxi, China) for establishment of the articular cartilage defect model. All animal experiments were performed in accordance with the NIH guide for the care and use of laboratory animals and approved by the Experimental Animal Ethics Committee of Guangxi Medical University (202204016). Following general anesthesia, an articular cartilage defect (4 mm in diameter and 3 mm in depth) was established and the rabbits were divided randomly into three groups. Rabbits in the sham group underwent joint exposure without defect implantation. Cells were digested and mixed with collagen hydrogel, then implanted into the defect site. Rabbits were intramuscular injected with penicillin for three consecutive days to prevent infection.

### Macroscopic evaluation and biomechanical test

At 4- and 8-week post-therapy, rabbits were sacrificed by intraperitoneal injection of overdose pentobarbital sodium and the joints were harvested. Joint surfaces were then exposure for macroscopic observation and the defect repair was scored according to the macroscopic score according to the study reported previously 39 by three researchers who were blinded to the grouping. Following macroscopic assessment, the biomechanics of the engineered cartilage tissue was tested by using a universal material testing machine (Instron, USA).

### Histochemical staining and score

Femurs and cells were fixed by immersing in 5% paraformaldehyde solution for 3 days, and then tissues were decalcified by solution containing 10% ethylenediaminetetraacetic acid (EDTA) for about two months. Tissue and cell samples were subsequently undergone dehydration, embedding and section. Sections were stained with hematoxylin-eosin (HE; Solarbio, Beijing, China), safranin O-fast green (Solarbio, Beijing, China) or toluidine blue (Solarbio, Beijing, China) according to the instructions of manufacture. Histological assessment of cartilage defect repair was performed using the International Cartilage Repair Society (ICRS) Histological Assessment Scale 40.

### Immunofluorescence and immunohistochemical staining

Sections of tissues and Cells were dewaxed to hydration, followed by incubating with 3% of H_2_O_2_ and goat serum successively for 10 min to block endogenous catalase and eliminate nonspecific staining. Then primary antibodies including COL2A1 (1:100, Proteintech, Wuhan, China), SOX9 (1:100, Proteintech, Wuhan, China), COL I (1:100, Proteintech, Wuhan, China), COLX (1:100, Proteintech, Wuhan, China), POU5F1 (1:100, Beyotime, Shanghai, China), AMPK (1:100, CST, USA), P-AMPK (1:100, CST, USA), T-mTOR (1:100, CST, USA), P-mTOR (1: 100, CST, USA) and BECN1 (1:100, Proteintech, Wuhan, China) were added and incubated at 4 °C overnight. Following staining with fluorescent secondary antibodies and DAPI, images were captured with a fluorescent microscope (Olympus, Japan) and semi-quantification analysis of fluorescence intensity was performed by using ImageJ software.

### Statistical analysis

The data we collected was analyzed by Independent Samples t-Test (for comparison of two groups) or One-way Analysis of Variance (ANOVA) (for comparison of three and more groups) using SPSS 22.0 software. Differences between groups for ANOVA were analyzed by LSD post hoc test. Data was exhibited as means ± standard deviation (SD). *p <* 0.05 was considered to be statistically significant.

## Supplementary Material

Supplementary figures.
