# Supplementary material for: Biomimetic ECM collagen hydrogel induces chondrogenic differentiation of BMSCs by activating autophagy through POU5F1-mediated AMPK/mTOR signaling pathway
Source: Theranostics. 2026 Mar 30;16(10):5770–87. doi: 10.7150/thno.126710 (PMC13081165; doi:10.7150/thno.126710)
Supplement: Supplementary file 1 — Supplementary figures. [file thnov16p5770s1.pdf]

Biomimetic ECM collagen hydrogel induces chondrogenic differentiation of BMSCs by activating autophagy through POU5F1-mediated AMPK/mTOR signaling pathway

Xiao Ru[1,2#], Zhongwen Yu[1, 3#], Jun Li[1, 2#], Fuben Xu[4], Guojie Xu[3], Junqi Xie[1,2], Simeng Yu[1, 2], Pan Hu[1, 2], YuanYuan Liu[1, 2], Li Zheng[1, 2]*, Jinmin Zhao[1, 3]*, Zhenhui Lu[1, 2]*

1 Guangxi Engineering Center in Biomedical Materials for Tissue and Organ Regeneration, Collaborative Innovation Centre of Regenerative Medicine and Medical BioResource Development and Application, Guangxi Key Laboratory of Regenerative Medicine, The First Affiliated Hospital of Guangxi Medical University, Nanning, Guangxi 530021, China
2 Life Sciences Institute, Guangxi Medical University, Nanning, Guangxi 530021, China
3 Department of Orthopaedics Trauma and Hand Surgery, The First Affiliated Hospital of Guangxi Medical University, Nanning, Guangxi 530021, China
4 Genetic and Metabolic Central Laboratory, Guangxi Birth Defects Research and Prevention Institute, Maternal and Child Health Hospital of Guangxi Zhuang Autonomous Region, Nanning, Guangxi 530003, China

Corresponding authors: zhengli@gxmu.edu.cn (L. Zheng), zhaojinmin@gxmu.edu.cn (J. Zhao) and zhenhuilu@sr.gxmu.cn (Z. Lu)

Xiao Ru, Zhongwen Yu, Jun Li and Fuben Xu contributed equally to this work.

Table S1 Primer sequences used in the RTq-PCR

**Gene names**	**Forward primer**	**Reverse primer**
BECN1	CCATTACTTGCCACAGCCCA	GACACCATCCTGGCGAGTTT
ATG5	AGCTGCATACACTTGGCGAT	CAGCTCAGGGTGATTCCGTT
ATG3	CATTTAGTCCACCACTGTCCC	CCATCCTCCATCACCATCATC
ATG7	ATAACGTCCTTCCCGTCAGC	CACTCAGACGGTCTCCTCGT
P62	TGACCAGATCCCAGAGTT	TGCACAAGAGTTCCGTAG
POU5F1	GTGTTCAGCCAAACCACCATC	CTCATTGTTGTCGGCCTCCT
SOX9	GCATGAGCGAGGTCCATTCC	AGGTACTGGTCGAACTCGTTG
ACAN	TTGCCTTTGTGGACACCAGT	GAGCCAAGGACGTAAACCCA
Col2a1	TGCAGGAGGGGAAGAGGTAT	GGCAGTCCTTGGTGTCTTCA
GAPDH	GTCATCATCTCAGCCCCCTC	GGATGCGTTGCTGACAATCT

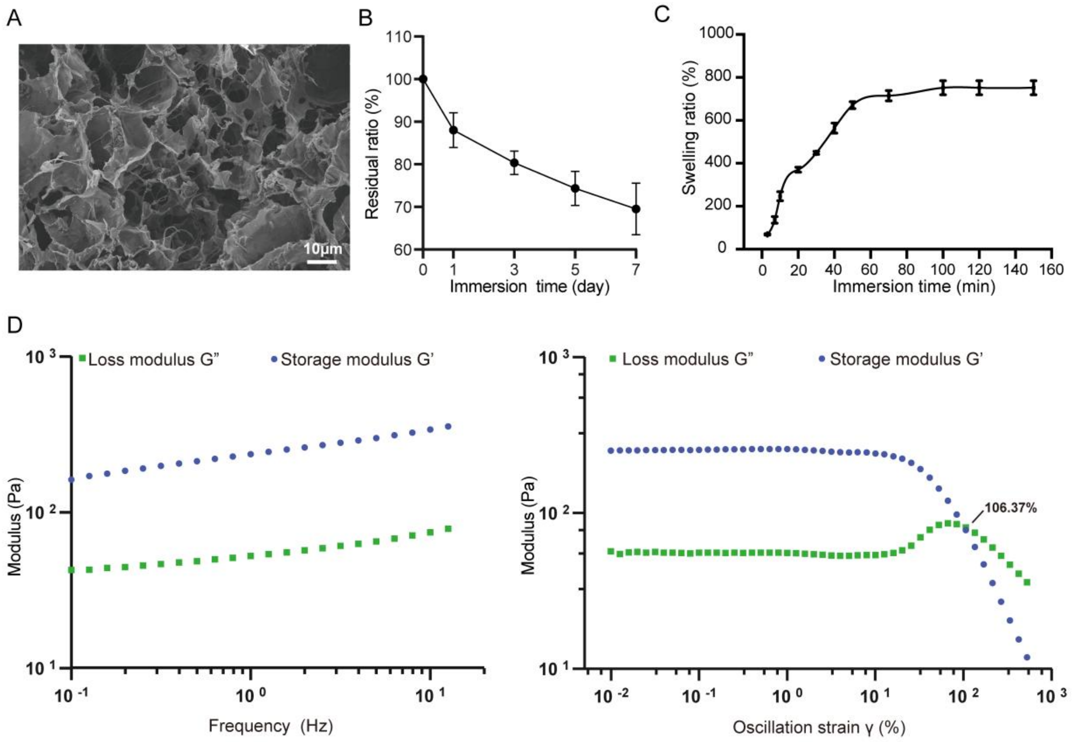

Figure S1 (A) SEM image, **(B)** residual ratio, **(C)** swelling ratio and **(D)** rheology of collagen hydrogel (Scale bar: 10 μm; Mean ± SD, n = 3).

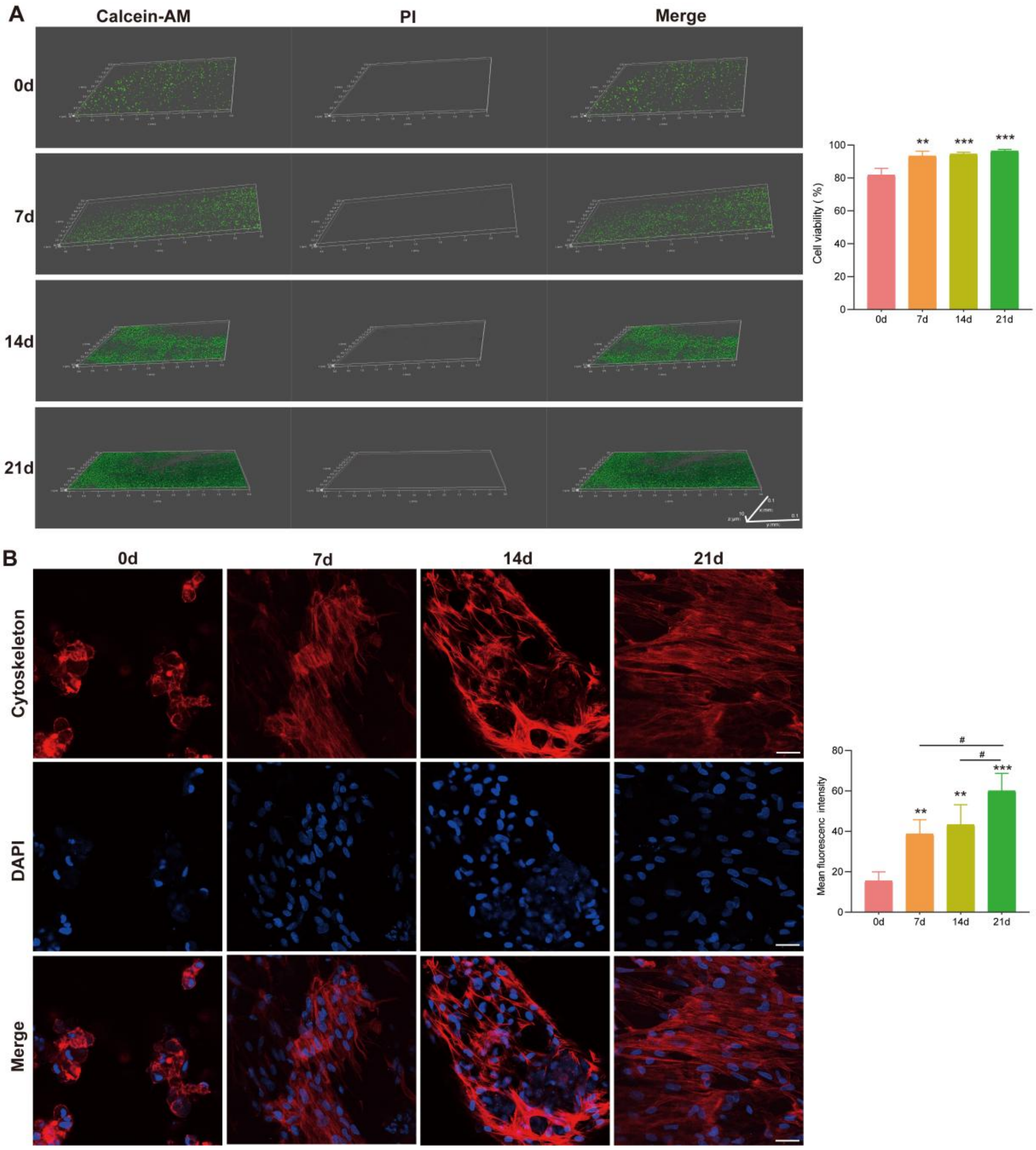

Figure S2 Proliferation of BMSCs cultured within 3D culture model. (A) Calcein-AM/PI staining and cell viability of BMSCs cultured within 3D culture model. (**B)** Cytoskeleton staining of BMSCs and semi-quantification analysis of fluorescence intensity (Scale bars: 100 μm; Mean ± SD, n=3; $^{**}p < 0.01$, $^{***}p < 0.001$ *vs.* day 0; $^{\#}p < 0.001$ for intergroup comparisons).

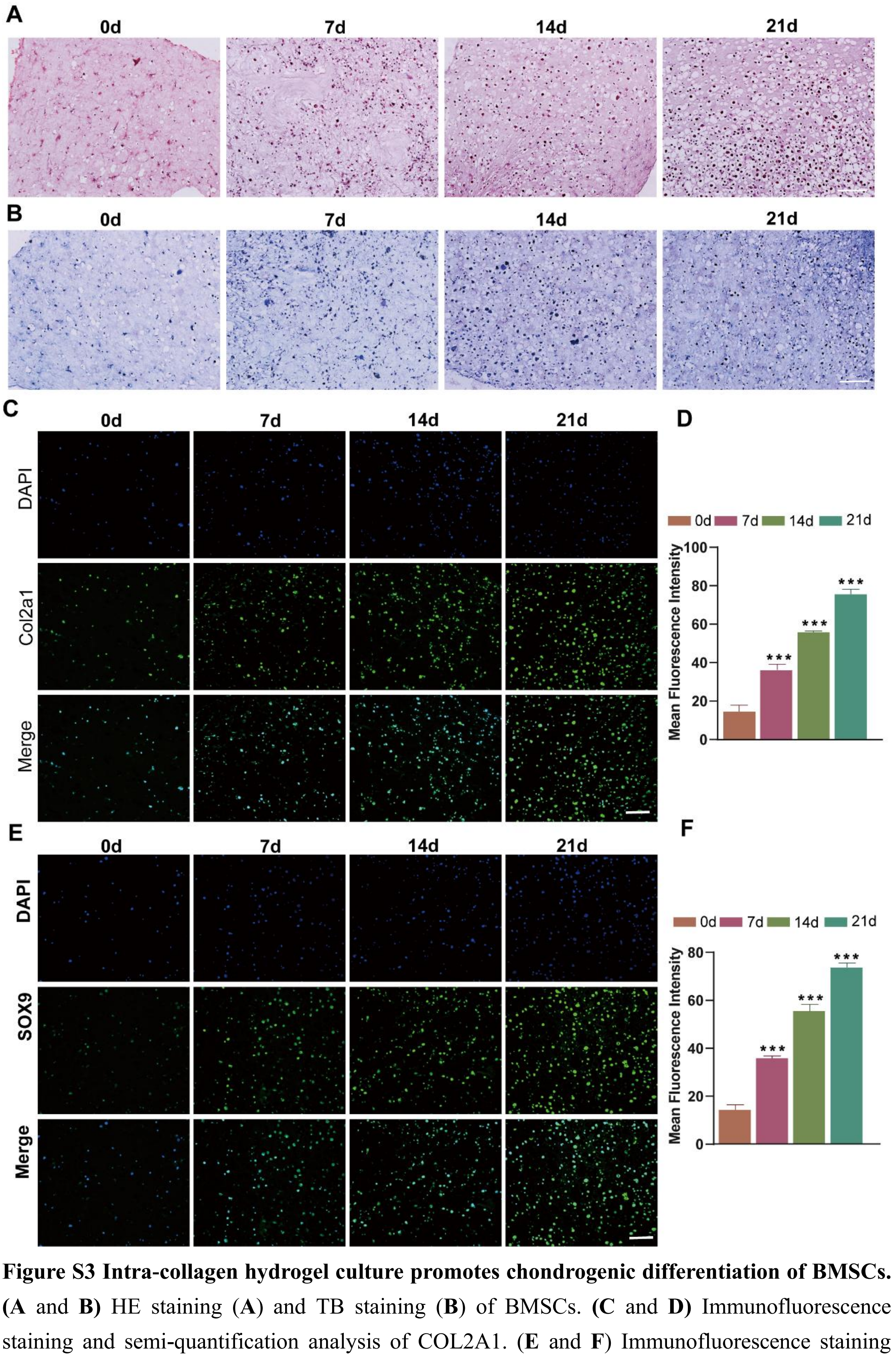

Figure S3 Intra-collagen hydrogel culture promotes chondrogenic differentiation of BMSCs. **(A** and **B)** HE staining (**A**) and TB staining (**B**) of BMSCs. **(C** and **D)** Immunofluorescence staining and semi-quantification analysis of COL2A1. (**E** and **F**) Immunofluorescence staining and semi-quantification analysis of SOX9 (Scale bars: 200 μm; Mean ± SD, n=3; ***$p < 0.001$ *vs.*day 0).

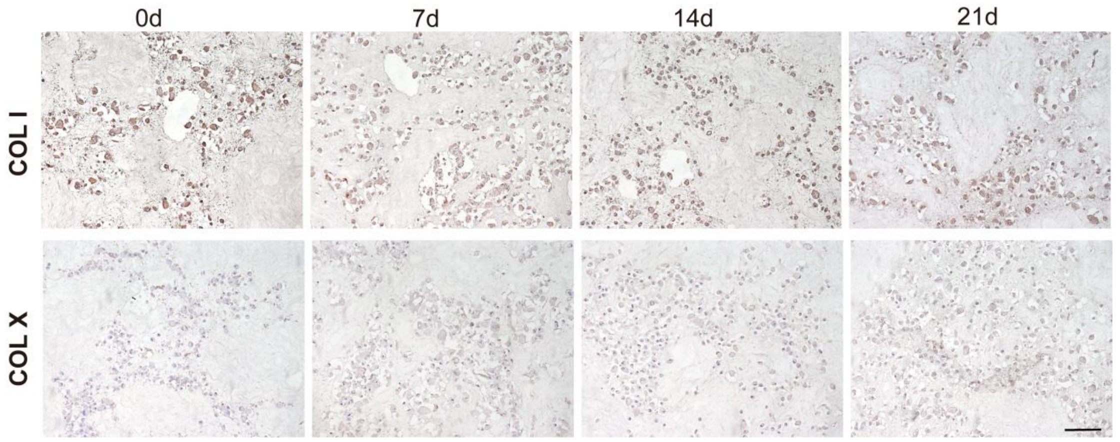

Figure S4 Immunohistochemical staining for COL I and COL X of BMSCs (Scale bar: 50 μm).

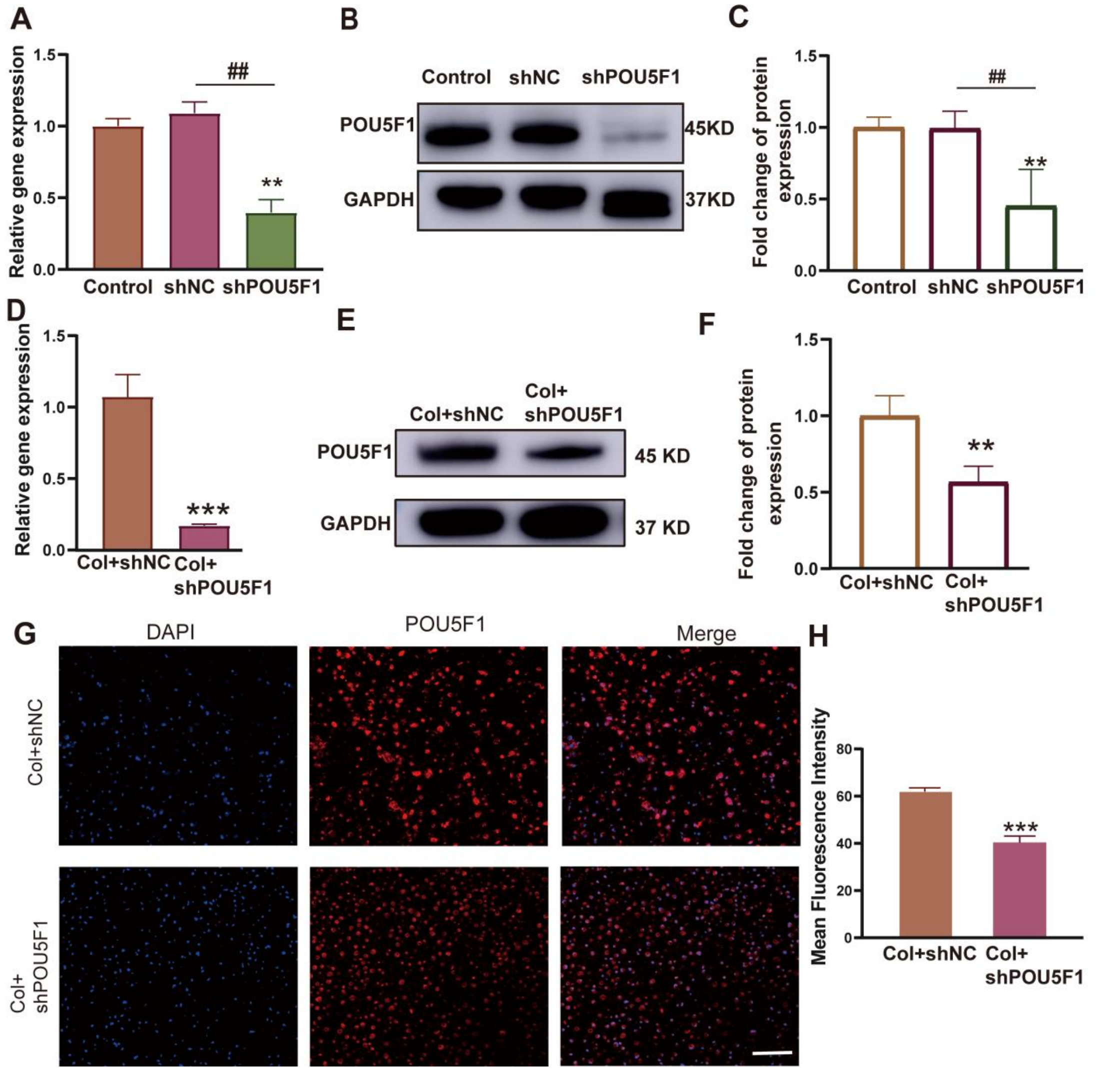

Figure S5 (**A**-**C**) Relative mRNA (**A**) and protein (**B**-**C**) expression levels of POU5F1 in the normal, shNC- or shPOU5F1- transfected BMSCs. (**D**-**H**) Relative mRNA (**D**) and protein (**E**-**H**) expression levels of POU5F1 in the shNC- or shPOU5F1-transfected BMSCs cultured within collagen hydrogel (Scale bar: 200 μm, Mean ± SD, n=3; $^{**}p<0.01$, $^{***}p<0.001$ *vs.*Control or Col+shNC; $^{\#\#}p<0.01$ for intergroup comparisons).
